# Effects of intraoperative fluid balance during pancreatoduodenectomy on postoperative pancreatic fistula: an observational cohort study

**DOI:** 10.1186/s12893-023-01978-9

**Published:** 2023-04-13

**Authors:** Le Zhang, Yuelun Zhang, Le Shen

**Affiliations:** 1 Department of Anesthesiology, Peking Union Medical College Hospital, Chinese Academy of Medical Sciences and Peking Union Medical College, Beijing, China; 2 Medical Research Center, Peking Union Medical College Hospital, Chinese Academy of Medical Sciences and Peking Union Medical College, Beijing, China; 3 State Key Laboratory of Complex Severe and Rare Diseases, Peking Union Medical College Hospital, Beijing, China

**Keywords:** Pancreaticoduodenectomy, Intraoperative fluid balance, Postoperative pancreatic fistula, Risk factors, Postoperative complications

## Abstract

**Background:**

Perioperative fluid management during major abdominal surgery has been controversial. Postoperative pancreatic fistula (POPF) is a critical complication of pancreaticoduodenectomy (PD). We conducted a retrospective cohort study to analyze the impact of intraoperative fluid balance on the development of POPF.

**Methods:**

This retrospective cohort study enrolled 567 patients who underwent open pancreaticoduodenectomy, and the demographic, laboratory, and medical data were recorded. All patients were categorized into four groups according to quartiles of intraoperative fluid balance. Multivariate logistic regression and restricted cubic splines (RCSs) were used to analyze the relationship between intraoperative fluid balance and POPF.

**Results:**

The intraoperative fluid balance of all patients ranged from -8.47 to 13.56 mL/kg/h. A total of 108 patients reported POPF, and the incidence was 19.0%. After adjusting for potential confounders and using restricted cubic splines, the dose‒response relationship between intraoperative fluid balance and POPF was found to be statistically insignificant. The incidences of bile leakage, postpancreatectomy hemorrhage, and delayed gastric emptying were 4.4%, 20.8%, and 14.8%, respectively. Intraoperative fluid balance was not associated with these abdominal complications. BMI ≥ 25 kg/m^2^, preoperative blood glucose < 6 mmol/L, long surgery time, and lesions not located in the pancreas were independent risk factors for POPF.

**Conclusion:**

The study did not find a significant association between intraoperative fluid balance and POPF. Well-designed multicenter studies are necessary to explore the association between intraoperative fluid balance and POPF.

## Background

From the perspective of anesthesiologists, perioperative fluid management is an important part of their anesthesia scheme. The fluid management strategies vary among anesthesiologists. In recent years, the classical perioperative fluid balance described in textbooks has been challenged. Fluid overload was reported to increase cardiopulmonary complications and impair the recovery of gastrointestinal function [1]. Restrictive fluid management has been thought to be beneficial for decreasing postoperative complications and improving prognosis for patients who underwent intraabdominal surgery [2]. A meta‑analysis suggested that patients receiving restricted fluid therapy were associated with rapid recovery and shorter length of hospital stay compared with liberal management during abdominal surgery [3]. In the consensus on the perioperative care of colorectal surgery patients, restrictive fluid management was recommended in the protocol of enhanced recovery after surgery (ERAS) [4]. Nevertheless, due to the limited quantity and quality of research, restrictive fluid management has been controversial during major abdominal surgery.

Pancreaticoduodenectomy (PD), as one of the most complex and traumatic abdominal operations, is associated with many postoperative abdominal complications, including pancreatic fistula, bile leak (BL), postpancreatectomy hemorrhage (PPH), delayed gastric emptying (DGE), and intra-abdominal infection [5]. Postoperative pancreatic fistula (POPF) is a critical complication with an incidence ranging from 10 to 34% [6]. If timely and effective treatment is not available, patients with POPF could experience intra-abdominal infection, intra-abdominal bleeding, and even death, consequently extending the length of hospital stay and exacerbating the medical burden [7].

We used to hold the opinion that whether POPF occurred depended largely on the technique and experience of surgeons. Some previous studies have clarified multiple perioperative factors related to POPF [8–10]. However, only a few studies have analyzed the correlation between intraoperative fluid management and POPF. Whether restrictive fluid management lowers the risk of POPF remains unclear. Therefore, we conducted a retrospective cohort study to clarify the impact of intraoperative fluid balance on the development of POPF. In addition, we performed exploratory analysis to investigate risk factors for POPF and the relationship between intraoperative fluid balance and other abdominal complications after PD.

## Methods

### Study design and patient

After approval by the institutional ethics board of Peking Union Medical College Hospital (K1522, 28 June 2022), the study created a retrospective cohort of patients who underwent open pancreatoduodenectomy between July 2016 and July 2021 at Peking Union Medical College Hospital. The surgeries were performed by an experienced surgical team. Indications for the surgery were tumors and other disorders of the pancreas, duodenum, bile duct, and adjacent other organs. The standard procedure of PD involved the removal of the pancreatic head, duodenum, first 15 cm of the jejunum, common bile duct, gallbladder, partial gastrectomy, and the reconstruction of gastrointestinal continuity. An end-to-side anastomosis was adopted in pancreaticojejunostomy, biliary-enteric anastomosis or gastrointestinal anastomosis. Modified PD, pylorus-preserving pancreaticoduodenectomy (PPPD) which preserves the gastric antrum, pylorus, and proximal 3 to 4 cm of the duodenum has been gradually used in the hospital for gastrointestinal continuity. The inclusion criteria were as follows: 1) older than 18 years; 2) ASA I–III; 3) patients who underwent elective open PD (including bowel resection, anastomosis, ostomy, partial vascular resection or reconstruction). The exclusion criteria were as follows: 1) patients who underwent total pancreatectomy and duodenectomy and 2) patients who lacked fluid records.

### Intraoperative fluid balance

According to the Chinese conventional infusion regimen, the intraoperative fluid volume was calculated by subtracting the total fluid output (preoperative loss during fasting, physiological required volume, urine output, blood loss, third space fluid loss) from the total fluid input (crystalloids, colloids, blood products). Specifically speaking, we used the “4–2-1” calculation to evaluate preoperative loss during fasting and physiological required volume which were essential for metabolism and fluid redistribution during the perioperative period. For example, A 60-kg patient would require (4 $\times$ 10)$+$(2 $\times$ 10)$+$(40 $\times$ 1)$=$ 100 mL/h maintenance water. As for preoperative fasting period, patients were instructed to abstain from food and water after 12 p.m. the night before surgery and most of them underwent the surgery at 8 a.m. the next day. The third space fluid loss was calculated by 6 mL/kg/h [11]. Intraoperative body fluid drainage like biliary drainage was not included in the fluid balance calculation for the volume could be ignored. Finally, the intraoperative fluid balance was equal to the intraoperative fluid volume divided by the patients’ weight and anesthesia time. The patients were categorized into 4 groups according to intraoperative fluid balance quartiles: Q1 ≤ -1.62, -1.61 < Q2 ≤ 0.80, -0.79 < Q3 ≤ 0.02, Q4 ≥ 0.03 mL/kg/h.

### Endpoints

The primary endpoint was clinically relevant POPF (CR-POPF: grade B $+$ C). In 2016, the International Study Group of Pancreatic Surgery (ISGPS) updated the POPF definition and grading system, which has been widely recognized and used. A clinically relevant POPF is defined as a drain output of any measurable volume of fluid with amylase level greater than 3 times the upper institutional normal serum amylase level, associated with a clinically relevant development/condition related directly to the POPF. Biochemical leakage no longer belonged to the category of POPF. Grade B was accompanied by a clinical change in the postoperative management or persistent drainage > 3 weeks, while Grade C required reoperation or led to organ failure or death [12]. The secondary endpoints included Grade B POPF, Grade C POPF, BL, PPH, and DGE. Likewise, the definitions of BL and PPH are derived from the ISGPS [13, 14]. Yeo et al. provided a clear definition of DGE in their prospective, randomized, and placebo-controlled trial. We chose to use the same definition for our study [15].

### Study variables

We collected demographic, laboratory, and medical data via the electronic medical record system and surgical anesthesia system of Peking Union Medical College Hospital. The baseline characteristics included age, sex, BMI, and comorbidities. BMI was expressed as a continuous variable as well as a categorical variable, and BMI was categorized into two groups: < 25 kg/m^2^, ≥ 25 kg/m^2^. The preoperative variables consisted of abdominal surgery history, lesions site, ASA classification, preoperative fluid infusion, and laboratory data (preoperative albumin, bilirubin and blood glucose). The grouping thresholds of the laboratory data were determined by the medical reference value. The intraoperative variables were comprised of the anesthetic mode, surgery option, surgery time, and intraoperative fluid management (blood loss, urine output, red blood cells, fresh frozen plasma, crystalloid/colloid volume). PD and PPPD were two surgical options.

### Statistical analyses

All participants were categorized into four groups according to quartiles of intraoperative fluid balance. Continuous variables were presented as the mean ± standard deviation or medians and interquartile ranges where appropriate. Categorical data were shown as numbers and percentages. Univariate and multivariate nonconditional logistic regression analyses were performed to assess the association between the fluid balance quartiles and primary outcome. Variables potentially associated with POPF or clinically important were selected for logistic regression. Odds ratios (ORs) and 95% confidence intervals (95% CIs) across the fluid balance quartiles (the lowest quartile as a reference) were calculated. Based on a multivariate logistic regression model, we used restricted cubic splines (RCSs) to explore the nonlinear dose‒response relationship between intraoperative fluid balance and POPF, with three knots defined at the 10th, 50th, and 90th percentiles of intraoperative fluid balance [16]. Data were analyzed using SPSS version 25.0 (SPSS, Inc., Chicago, IL, USA). The restricted cubic spline analysis and the related figure were conducted with R version 4.2.0 (R Foundation for Statistical Computing, Vienna, Austria) using the rms and ggplot2 packages. All tests were 2-sided, and all tests were conducted at the 5% significance level.

## Results

### Demographics

**Fig. 1:**
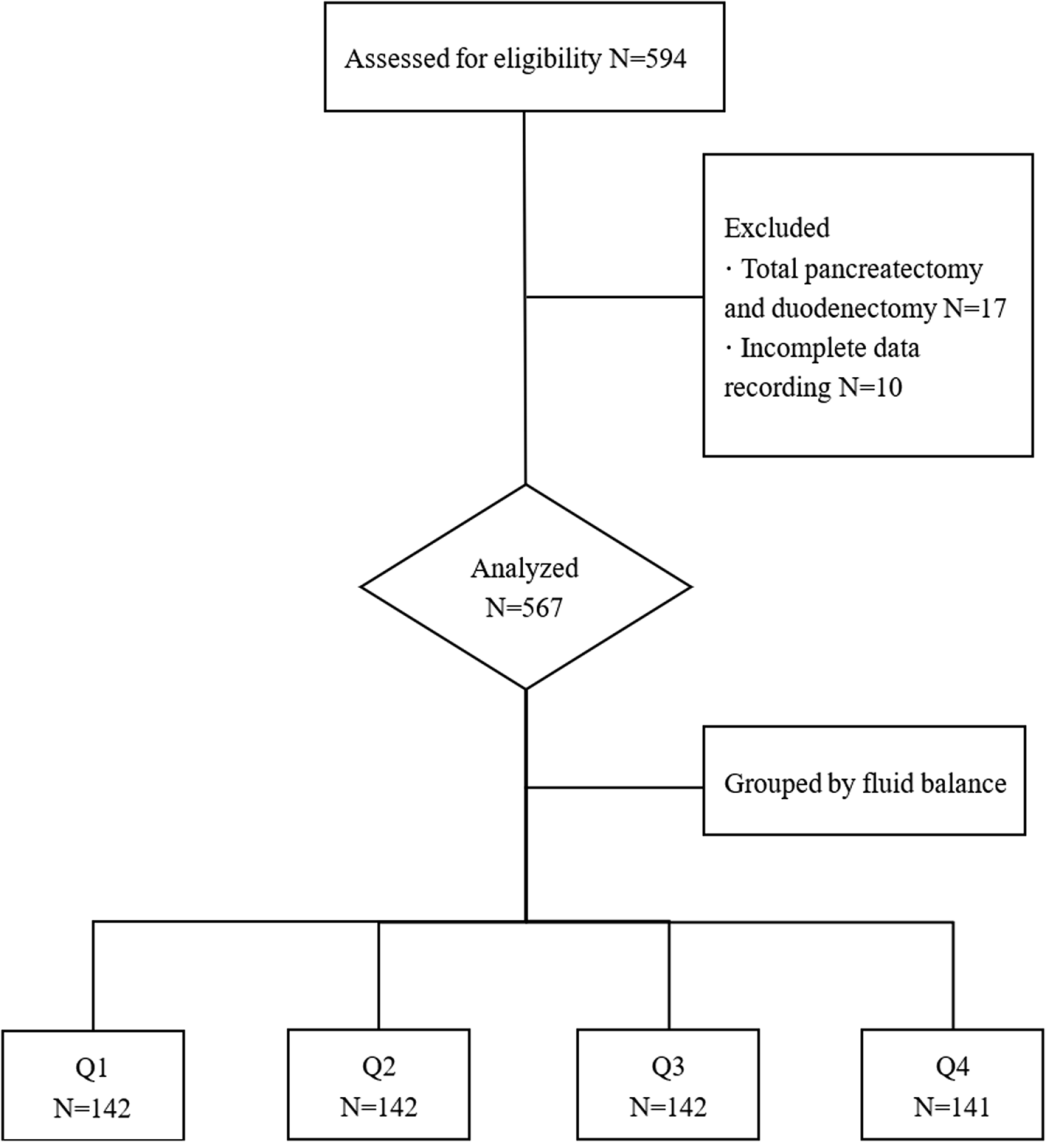
Patient enrollment flowchart. fluid balance: Q1 ≤ -1.62, -1.61 < Q2 ≤ 0.80, -0.79 < Q3 ≤ 0.02, Q4 ≥ 0.03 mL/kg/h

We collected 595 patients who underwent elective open PD between July 2016 and July 2021 at Peking Union Medical College Hospital, and 567 patients were finally included for statistical analysis. Patient enrollment flowchart was shown in Fig. 1. A total of 336 (59.3%) of the patients were male. The patients were divided by their intraoperative fluid balance, ranging from -8.47 to 13.56 mL/kg/h, according to the quartiles. The demographic characteristics based on the four groups are shown in Table 1. The mean age of the enrolled patients was 58.68 ± 10.87 years old, and there was no significant difference in the patients’ ages among the groups. As the intraoperative fluid balance increased, the proportion of overweight patients decreased.

**Table 1:** Cohort characteristics by fluid balance quartiles

Variables	All patients	Q1	Q2	Q3	Q4	*P*
	**(*n* = 567)**	**(*n* = 142)**	**(*n* = 142)**	**(*n* = 142)**	**(*n* = 141)**	
**Baseline characteristics**						
**Age, mean (SD), years**	58.7 ± 10.9	58.5 ± 10.2	58.4 ± 10.4	58.8 ± 10.7	59.0 ± 12.2	0.967
**Sex, male, n (%)**	336 (59.3%)	77 (54.2%)	100 (70.4%)	84 (59.2%)	75 (53.2%)	0.012*
**BMI, mean (SD), kg/m**^**2**^	22.8 ± 3.3	24.3 ± 3.1	23.5 ± 3.0	22.5 ± 3.1	20.7 ± 2.9	< 0.001*
**BMI ≥ 25 kg/m**^**2**^**, n (%)**	134 (23.6%)	53 (37.3%)	39 (27.5%)	29 (20.4%)	13 (9.2%)	< 0.001*
**Comorbidities**						
**Smoking history, n (%)**	214 (37.7%)	48 (33.8%)	64 (45.1%)	56 (39.4%)	46 (32.6%)	0.116
**Alcohol intake, n (%)**	179 (31.6%)	44 (31%)	55 (38.7%)	40 (28.2%)	40 (28.4%)	0.184
**Hypertension, n (%)**	168 (29.6%)	53 (37.3%)	46 (32.4%)	43 (30.3%)	26 (18.4%)	0.004*
**Diabetes mellitus, n (%)**	128 (22.6%)	35 (24.6%)	34 (23.9%)	31 (21.8%)	28 (19.9%)	0.765
**Coronary artery disease, n (%)**	44 (7.8%)	12 (8.5%)	9 (6.3%)	15 (10.6%)	8 (5.7%)	0.417
**Preoperative characteristics**						
**Abdominal surgery history, n (%)**	164 (28.9%)	38 (26.8%)	39 (27.5%)	36 (25.4%)	51 (36.2%)	0.176
**Lesion site, pancreas, n (%)**	356 (62.8%)	90 (63.4%)	90 (63.4%)	91 (64.1%)	85 (60.3%)	0.913
**ASA classification**						0.652
**Class I–II, n (%)**	477 (84.1%)	120 (84.5%)	122 (85.9%)	121 (85.2%)	114 (80.9%)	
**Class III, n (%)**	90 (15.9%)	22 (15.5%)	20 (14.1%)	21 (14.8%)	27 (19.1%)	
**Pre-ALB, mean (SD), g/L**	40 ± 4.8	40.9 ± 4.4	40.1 ± 4.3	39.6 ± 4.9	39.3 ± 5.3	0.020*
**Pre-ALB < 35 g/L, n (%)**	78 (13.8%)	10 (7%)	12 (8.5%)	25 (17.6%)	31 (22%)	< 0.001*
**Pre-TBIL, median (IQR), μmol/L**	18.6 (11.4 ~ 69.5)	17.9 (11.5 ~ 67.7)	18.8 (11.8 ~ 72.9)	19.6 (11.3 ~ 64.9)	18.5 (11.1 ~ 88.8)	0.918
**Pre-TBIL > 17.1 μmol/L, n (%)**	303 (53.4%)	74 (52.1%)	75 (52.8%)	78 (54.9%)	76 (53.9%)	0.967
**Pre-GLU, mean (SD), mmol/L**	6.5 ± 2.4	6.4 ± 2.2	6.5 ± 2.0	6.8 ± 2.9	6.5 ± 2.4	0.384
**Pre-GLU > 6 mmol/L, n (%)**	244 (43.0%)	57 (40.1%)	69 (48.6%)	61 (43%)	57 (40.4%)	0.446
**Preoperative fluid infusion, n (%)**	200 (35.3%)	32 (22.5%)	42 (29.6%)	55 (38.7%)	71 (50.4%)	< 0.001*
**Intraoperative characteristics**						
**TIVA, n (%)**	50 (8.8%)	11 (7.7%)	9 (6.3%)	9 (6.3%)	21 (14.9%)	0.030*
**Surgery time, mean (SD), h**	5.9 ± 1.4	5.9 ± 1.3	6.0 ± 1.4	5.9 ± 1.4	5.7 ± 1.4	0.273
**PPPD, n (%)**	76 (13.4%)	20 (14.1%)	24 (16.9%)	20 (14.1%)	12 (8.5%)	0.211
**Blood transfusion, n (%)**	301 (53.1%)	40 (28.2%)	64 (45.1%)	84 (59.2%)	113 (80.1%)	< 0.001*
**Blood loss, median (IQR), L**	0.5 (0.3 ~ 0.7)	0.4 (0.3 ~ 0.6)	0.4 (0.3 ~ 0.7)	0.4 (0.3 ~ 0.8)	0.5 (0.4 ~ 0.9)	0.006*
**RBC, median (IQR), units**	0 (0 ~ 3)	0 (0 ~ 0)	0 (0 ~ 2)	2 (0 ~ 2)	4 (1 ~ 4)	< 0.001*
**FFP, median (IQR), L**	0 (0 ~ 0.4)	0 (0 ~ 0.05)	0 (0 ~ 0.4)	0.2 (0 ~ 0.4)	0.4 (0 ~ 0.4)	< 0.001*
**Crystalloid, median (IQR), L**	3 (2.4 ~ 3.7)	2.45 (2.1 ~ 2.7)	2.85 (2.5 ~ 3.4)	3.3 (2.7 ~ 3.9)	3.4 (2.8 ~ 4.3)	< 0.001*
**Colloid, median (IQR), L**	1 (0.5 ~ 1)	0.5 (0.5 ~ 1)	1 (0.5 ~ 1)	1 (0.5 ~ 1)	1 (0.5 ~ 1.5)	< 0.001*

*Pre* Preoperative, *SD* Standard deviation, *IQR* Interquartile range, *ALB* Albumin, *TBIL* Total bilirubin, *GLU* Blood glucose, *TIVA* Total intravenous anesthesia, *PPPD* Pylorus-preserving pancreaticoduodenectomy, *RBC* Red blood cell, *FFP* Fresh frozen plasma

^*^P < 0.05

### Intraoperative fluid balance and POPF

**Fig. 2:**
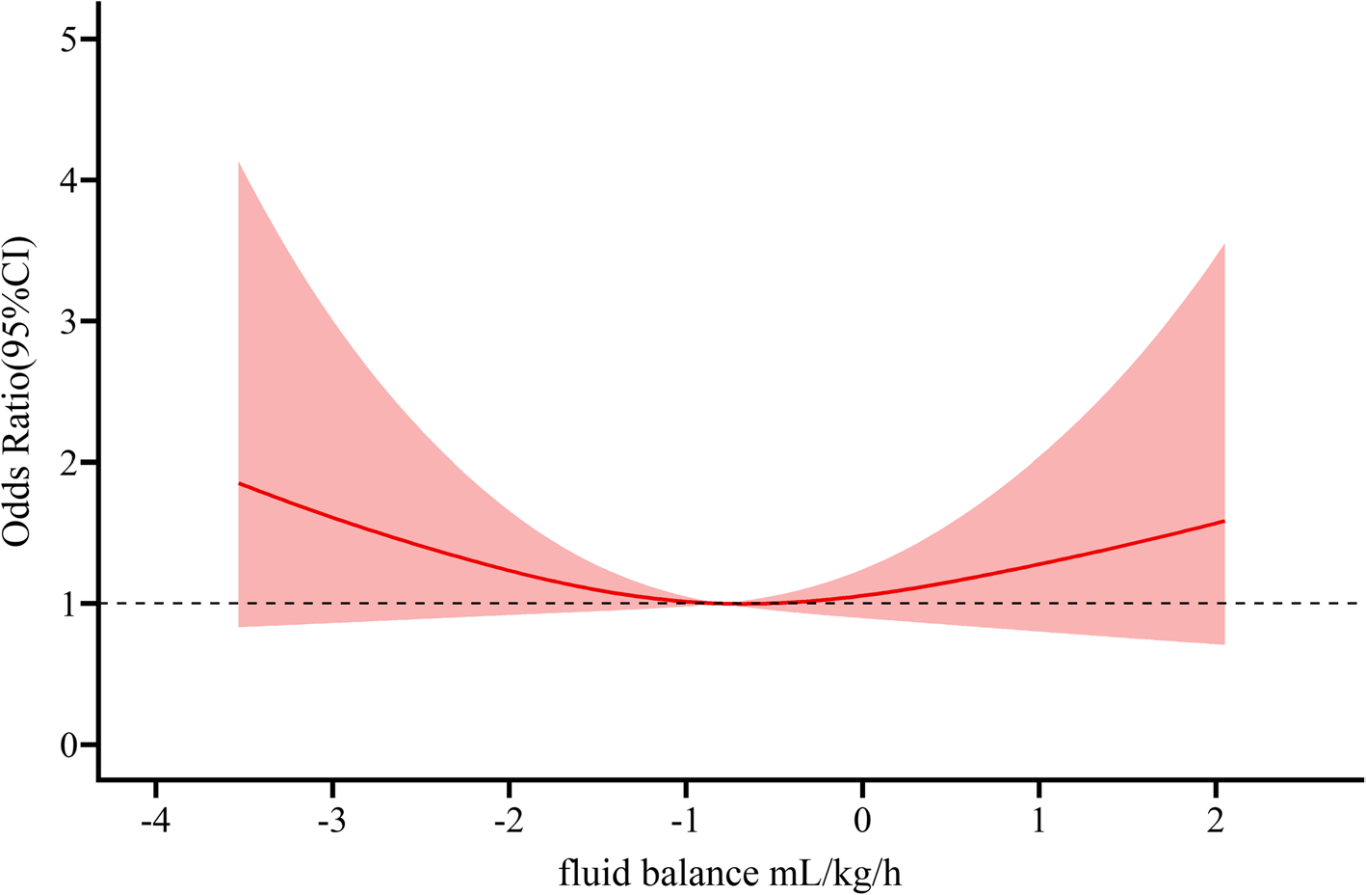
Adjusted ORs of postoperative pancreatic fistula according to intraoperative fluid balance. Odds ratios and 95% confidence intervals derived from restricted cubic spline regression, with knots placed at the 10th, 50th, and 90th percentiles of intraoperative fluid balance

Among the 567 patients, 108 were diagnosed with POPF, and the incidence was 19.0%. Among them, 80 patients were categorized into Grade B while 28 patients belonged to Grade C. There were 22 patients with POPF in the Q3 group of intraoperative fluid balance, which was lower than the other three groups. First, we used intraoperative fluid balance as a categorical variable and the lowest quartile as the reference to conduct a univariate analysis. The results showed no statistically significant relationship between intraoperative fluid balance and POPF. Then, multivariate logistic regression was performed after adjusting for potential confounding factors, including age, sex, BMI, abdominal surgery history, preoperative bilirubin, preoperative blood glucose, ASA classification, anesthetic mode, surgery option, and lesion site. The outcome did not change. Furthermore, we took into account the differentiation between grade B and grade C POPF, the outcome kept the same. The results are summarized in Table 2. Furthermore, we adjusted for the same potential confounders and modeled the association between intraoperative fluid balance and POPF using restricted cubic splines. The AIC value for the model with a 3-knot RCS function was less than those for the models with a 4-knot or 5-knot, suggesting that the model with a 3-knot RCS function was more adequate. The results are presented in Fig. 2. According to the OR curve, when the intraoperative fluid balance level was approximately -0.7 mL/kg/h, the risk of POPF was minimal. When the intraoperative fluid balance level became more positive or negative, the risk of POPF increased. Nevertheless, the *P* value of the test for the overall association between intraoperative fluid balance and POPF was insignificant, which meant that regardless of the shape of this curve, there was no significant association between intraoperative fluid balance and POPF.

**Table 2:** Associations between fluid balance and outcomes

	**Q1**	**Q2**	**Q3**	**Q4**	***P***
	**(*n* = 142)**	**(*n* = 142)**	**(*n* = 142)**	**(*n* = 141)**	
**Primary Outcome**					
**POPF, n (%)**	29 (20.42%)	29 (20.42%)	22 (15.49%)	28 (19.86%)	
**OR95%CI**	1	1 (0.56,1.78)	0.71 (0.39,1.32)	0.97 (0.54,1.73)	0.668
**Adjusted OR95%CI**^**†**^	1	1.15 (0.62,2.11)	0.86 (0.45,1.63)	1.3 (0.69,2.46)	0.618
**Secondary Outcomes**					
**Grade B POPF, n (%)**	21 (14.79%)	24 (16.90%)	15 (10.56%)	20 (14.18%)	
**Adjusted OR95%CI**^**†**^	1	1.38 (0.7,2.72)	0.92 (0.44,1.92)	1.31 (0.63,2.7)	0.62
**Grade C POPF, n (%)**	11 (7.75%)	4 (2.82%)	5 (3.5%)	8 (5.63%)	
**Adjusted OR95%CI**^**†**^	1	0.68 (0.22,2.08)	0.73 (0.23,2.27)	1.25 (0.42,3.69)	0.698
**BL, n (%)**	7 (4.9%)	5 (3.5%)	7 (4.9%)	6 (4.3%)	
**Adjusted OR95%CI**^**†**^	1	0.61 (0.18,2.02)	0.99 (0.33,2.98)	0.95 (0.29,3.06)	0.841
**PPH, n (%)**	27 (19%)	29 (20.40%)	25 (17.60%)	37 (26.20%)	
**Adjusted OR95%CI**^**†**^	1	1.15 (0.62,2.12)	0.97 (0.52,1.81)	1.86 (1.01,3.43)	0.115
**DGE, n (%)**	24 (16.90%)	14 (9.90%)	22 (15.50%)	24 (17.00%)	
**Adjusted OR95%CI**^**†**^	1	0.55 (0.27,1.13)	0.99 (0.51,1.89)	1.22 (0.63,2.36)	0.187

^**†**^adjusted by age, sex, *BMI*, abdominal surgery history, preoperative bilirubin, preoperative blood glucose, *ASA* classification, anesthetic mode, surgery option, and location of lesion. the lowest quartile as the reference. *POPF* Postoperative pancreatic fistula, *BL* Bile leakage, *PPH* Postpancreatectomy hemorrhage, *DGE* Delayed gastric emptying, *OR* Odds ratio, *CI* Confidence interval

### Other complications and risk factors for POPF

The incidences of BL, PPH, and GED were 4.4%, 20.8%, and 14.8%, respectively. We found that the association between intraoperative fluid balance and these abdominal complications was not statistically significant. For risk factors for POPF, after univariate regression analysis, we included the variables with *P* < 0.1 in the multivariate regression analysis, and the results are shown in Table 3. We found that BMI, preoperative blood glucose, surgery time, and lesion site were related to POPF. In contrast to the patients without POPF, the surgeries (OR: 1.29, 95% CI: 1.08–1.55) were longer, and the proportion of overweight patients (OR: 2.45, 95% CI: 1.54–3.91) was higher in the group of patients who developed POPF. However, preoperative hyperglycemia (blood glucose > 6 mmol/L, OR: 0.61, 95% CI: 0.38–0.97) and lesions located in the pancreas (OR: 0.43, 95% CI: 0.27–0.67) were protective factors for POPF.

**Table 3:** Risk factors for POPF based on logistic regression analysis

	**Univariate**		**Multivariate**	
	**OR95%CI**	***P* value**	**OR95%CI**	***P* value**
**BMI ≥ 25 kg/m**^**2**^	2.54 (1.62,3.98)	< 0.001	2.45 (1.54,3.91)	< 0.001
**Preoperative GLU > 6 mmol/L**	0.6 (0.39,0.94)	0.025	0.61 (0.38,0.97)	0.038
**Surgery time, h**	1.26 (1.09,1.46)	< 0.001	1.29 (1.08,1.55)	0.006
**Blood loss, L**	1.37 (1.01,1.87)	0.046	1.09 (0.75,1.58)	0.659
**Preoperative fluid infusion**	0.62 (0.39,0.99)	0.043	0.73 (0.45,1.19)	0.208
**Lesion site, pancreas**	0.45 (0.3,0.69)	< 0.001	0.43 (0.27,0.67)	< 0.001

*GLU* Blood glucose, *OR* Odds ratio, *CI* Confidence interval

## Discussion

Fluid management is complex and variable. Some studies tend to support restrictive fluid management, but the controversy over fluid management has never ceased. Zhang et al. enrolled 301 patients for a retrospective study, and the results showed that higher postoperative fluid balance appeared to be associated with morbidity after PD, systemic inflammatory response syndrome (SIRS), acute respiratory distress syndrome (ARDS), bleeding, and heart failure [17]. However, Meyhoff et al. performed an international, randomized trial among adult patients with septic shock in the ICU and found that intravenous fluid restriction did not decrease mortality at 90 days compared with standard intravenous fluid therapy [18].

The relationship between intraoperative fluid balance and the specific abdominal complication of POPF is also ambiguous. Some studies have demonstrated that the incidence of POPF in the high fluid volume group was higher than that in the low fluid volume group during PD, and intraoperative fluid excess was also considered a risk factor for POPF [19–23]. Nevertheless, in 2017, a meta-analysis evaluated the relationship between the perioperative fluid balance and postoperative complications of PD (including POPF), and no significant correlation was found [24]. Similarly, a meta-analysis published in 2018 demonstrated that intraoperative fluid balance did not affect the occurrence of POPF [25]. Interestingly, a retrospective cohort study showed that intraoperative fluid balance did not affect the incidence of POPF, but the higher cumulative fluid balance per body weight (FBPBW) at postoperative day 3 was an independent risk factor for CR-POPF [26]. Other studies have also demonstrated that patients with a high postoperative fluid balance presented a higher incidence of POPF [27–29]. Although the related mechanisms have not been thoroughly studied, there are some plausible claims. Trauma of the pancreatic surface in the PD procedure and leakage of a pancreatic-enteric anastomosis are the main reasons for POPF [12]. Fluid overload could cause pancreatic parenchyma and intestinal edema, increase the distance between capillaries and cells, impair gas exchange, and then lead to dysfunction or disruption of the pancreatojejunostomy [30, 31].

The study consecutively enrolled 567 patients who underwent PD surgery, and they were divided into four groups by the quartiles of intraoperative fluid balance to explore any difference in POPF between the four groups. Then, intraoperative fluid balance was regarded as a continuous variable, and we used restricted cubic splines to analyze whether a dose‒response relationship existed between intraoperative fluid balance and POPF. Finally, the relationship was found to be statistically insignificant. In addition, we did not find an association between intraoperative fluid balance and BL, DEG, or PPH. Identically, Braga et al. grouped patients undergoing PD according to whether they received a comprehensive ERAS protocol or standard perioperative care. A comprehensive ERAS protocol contained many items, such as a lack of bowel preparation and shortening of the preoperative fasting period, and the intravenous fluid infusion rate was lower in the ERAS group. The results showed that the incidence of POPF was similar in the two groups, as were BL, DGE, and PPH [32].

Sex, BMI, fasting blood glucose level, pancreatic texture, pancreatic duct, pancreatojejunostomy anastomosis technique, and tumor location were related risk factors for POPF. Our study found that BMI ≥ 25 kg/m^2^, preoperative hypoglycemia (blood glucose ≤ 6 mmol/L), long surgery time, and lesion not located in the pancreas were risk factors for POPF. Preoperative hypoglycemia, lesions not located in the pancreas, and BMI might lead to a softer pancreas, thereby influencing the occurrence of POPF [7–10]. In addition, when the operation was complicated and difficult, the surgery took longer, and there was a tendency to develop related postoperative complications. As the most critical complication of PD, the treatment and prevention of POPF have been the main concerns for pancreatic surgeons. Besides early identification of related risk factors for POPF, other contemporary mitigation strategies include the use of abdominal drains, prophylactic somatostatin analogues, modified anastomotic techniques, and anastomotic stents [33]. Recently, a study showed that coronary artery stent (CAS) positioning in pancreatico-jejunal anastomosis could be a novel mitigation strategy in the prevention of POPF [34].

The definition of POPF is relatively uniform in many studies, but the calculation of perioperative fluid balance or fluid management varies. There is no uniform standard for the definition of perioperative fluid balance or fluid management, especially for the cutoff values, which can vary [20–23], and this makes it difficult to integrate and summarize the results of current clinical studies. Relevant research results should be treated with caution. Many anesthesiologists still use the conventional infusion regimen described in textbooks when formulating plans before major surgery. Our definition of fluid balance was based on that. However, the conventional infusion regimen took the patient’s perioperative physiological needs and the third space fluid loss into account; patients were thought to be relatively hypovolemic and therefore required relatively more aggressive fluid replacement. This explains why many patients received a negative intraoperative balance in our study. Our definition of intraoperative fluid balance consisted of the surgery time and patient weight, which helped to reduce the impact of the surgery time and patient weight on intraoperative fluid balance. The volume of intraoperative RBC and colloid infusion in Q3 and Q4 was higher than that in Q1 and Q2. However, intraoperative fluid balance was an integrated variable, and it was difficult for us to explain whether the intraoperative RBC and colloid infusion affected the incidence of POPF. Moreover, we did not consider the difference in the volume effect of crystalloids and colloids when calculating the intraoperative fluid balance.

The anesthesiologists in our institution always formulate the intraoperative fluid management plans in advance based on the patient’s physiological needs. In fact, the intraoperative fluid management is affected by many factors, such as intraoperative bleeding, patient cardiovascular function, depth of anesthesia, and use of vasoactive drugs in a major surgery such as PD. Therefore, many anesthesiologists often use invasive monitoring methods in combination with other hemodynamic parameters to evaluate the patient's volume status and adjust their fluid management plan in a timely manner. As a result, the concept of goal-directed fluid therapy (GDFT) may be the most reasonable fluid management strategy available.

### Limitation

The present study had some limitations. First, this study analyzed the association between intraoperative fluid balance and the incidence of POPF but did not further analyze the effect of intraoperative fluid balance on the severity of POPF. Second, confounding factors such as pancreatic texture and pancreatic duct played an influential role in whether pancreatic fistula occurred. However, we collected data retrospectively and the operative recordings in the electronic medical record system seldom documented these variables. The association between intraoperative fluid balance and the development of POPF could be more reliable if adjusted by these related factors. Third, all the patients were treated at a single center. To validate the effect of intraoperative fluid balance on POPF, a multicenter study with a larger number of patients is needed in the future.

## Conclusions

Our study failed to find a significant association between intraoperative fluid balance and POPF. Furthermore, the correlations between fluid balance and other abdominal complications were statistically insignificant. On the other hand, the findings of this study indicated that BMI, blood glucose, surgery time and lesion site were closely related to POPF. It is necessary to unify the definition of fluid balance and develop well-designed studies to validate the optimal fluid strategy and reduce abdominal complications such as POPF. Until then, the intraoperative fluid management plan of PD should be individualized and adjusted according to the actual situation.

## Data Availability

The datasets generated and/or analyzed during the current study are not publicly available due to the fear of improper use but are available from the corresponding author on reasonable request.

## Abbreviations

POPF: Postoperative pancreatic fistula
PD: Pancreaticoduodenectomy
RCS: Restricted cubic spline
ERAS: Enhanced recovery after surgery
BL: Bile leak
PPH: Postpancreatectomy hemorrhage
DGE: Delayed gastric emptying
PPPD: Pylorus-preserving pancreaticoduodenectomy
ISGPS: International study group of pancreatic surgery
OR: Odds ratio
CI: Confidence interval
SIRS: Systemic inflammatory response syndrome
ARDS: Acute respiratory distress syndrome
FBPBW: Cumulative fluid balance per body weight
CAS: Coronary artery stent
GDFT: Goal-directed fluid therapy
